# Recoupling the Cardiac Nitric Oxide Synthases: Tetrahydrobiopterin Synthesis and Recycling

**DOI:** 10.1007/s11897-012-0097-5

**Published:** 2012-06-19

**Authors:** Matthew S. Alkaitis, Mark J. Crabtree

**Affiliations:** 1 Nuffield Department of Clinical Laboratory Sciences, University of Oxford, John Radcliffe Hospital, Oxford, UK; 2 Laboratory of Malaria and Vector Research, National Institute of Allergy and Infectious Diseases, National Institutes of Health, Bethesda, MD USA; 3 Department of Cardiovascular Medicine, University of Oxford, John Radcliffe Hospital, Oxford, UK

**Keywords:** Nitric oxide, Nitric oxide synthase, Uncoupling, Superoxide, Tetrahydrobiopterin, Dihydrobiopterin, Sepiapterin, Sapropterin, GTP cyclohydrolase I, Dihydrofolate reductase, Endothelial cell, Cardiomyocyte, Hypertension, Heart failure, Atrial fibrillation, Ischemia, Reperfusion, Vasodilation, L-Arginine, Monomethylarginine, Asymmetric dimethylarginine, S-Glutathionylation, Peroxynitrite, Oxidative stress, NADPH oxidase

## Abstract

Nitric oxide (NO), a key regulator of cardiovascular function, is synthesized from L-arginine and oxygen by the enzyme nitric oxide synthase (NOS). This reaction requires tetrahydrobiopterin (BH4) as a cofactor. BH4 is synthesized from guanosine triphosphate (GTP) by GTP cyclohydrolase I (GTPCH) and recycled from 7,8-dihydrobiopterin (BH2) by dihydrofolate reductase. Under conditions of low BH4 bioavailability relative to NOS or BH2, oxygen activation is “uncoupled” from L-arginine oxidation, and NOS produces superoxide (O_2_^−^) instead of NO. NOS-derived superoxide reacts with NO to produce peroxynitrite (ONOO^−^), a highly reactive anion that rapidly oxidizes BH4 and propagates NOS uncoupling. BH4 depletion and NOS uncoupling contribute to overload-induced heart failure, hypertension, ischemia/reperfusion injury, and atrial fibrillation. L-arginine depletion, methylarginine accumulation, and S-glutathionylation of NOS also promote uncoupling. Recoupling NOS is a promising approach to treating myocardial and vascular dysfunction associated with heart failure.

## Introduction

Synthesis of the gaseous signaling molecule nitric oxide (NO) by the nitric oxide synthase (NOS) family of enzymes is a key mechanism of cardiovascular homeostasis. Three NOS isoforms, neuronal (nNOS), inducible (iNOS), and endothelial (eNOS), catalyze the reaction of molecular oxygen with the amino acid substrate L-arginine to produce L-citrulline and NO [1]. During this reaction, electrons donated by nicotinamide adenine dinucleotide phosphate (NADPH) at the carboxy-terminal reductase domain of NOS are passed to the heme catalytic center of the oxidase domain, where activation of molecular oxygen is “coupled” to NO synthesis by two successive monooxygenations of L-arginine [2]. The cofactor 6R-5,6,7,8-tetrahydrobiopterin (BH4) is required for these reactions; in its absence, electron flow to molecular oxygen becomes “uncoupled” from L-arginine oxidation, resulting in production of superoxide (O_2_^−^) instead of NO [3–6]. The combination of increased oxidative stress and impaired NO signaling resulting from NOS uncoupling has been implicated in the pathogenesis of a wide range of disease states, including atherosclerosis [7], hypertension [8, 9] and diabetes [10]. Advances in understanding the role of NO signaling in myocardial function have encouraged recent research into whether NOS uncoupling contributes to the pathogenesis of several aspects of heart failure. In this review, we will discuss current progress in this field, focusing on key studies that have identified BH4 bioavailability as a critical biochemical determinant of NOS uncoupling.

## BH4 Synthesis is a Determinant of NOS Uncoupling

### BH4 as a NOS Cofactor

Tetrahydrobiopterin was initially recognized as a cofactor required for enzymatic hydroxylation of phenylalanine, tyrosine, and tryptophan. Subsequently, the identity of the vasodilator known as “endothelial-derived relaxing factor” was discovered to be NO [11, 12], and BH4 also was found to be essential for NOS-mediated NO synthesis [13, 14]. As an NOS cofactor, BH4 performs both structural and biochemical functions. Structurally, BH4 stabilizes the active NOS homodimer [15, 16] and induces conformational changes that promote catalytic activity [17]. Biochemically, the donation of an electron by BH4 to produce a transient BH4^•^^+^ radical is required for the oxidation of L-arginine to L-citrulline [18–20] and associated formation of a ferrous iron-NO complex at the NOS heme catalytic center [1, 2]. In the final step of NO synthesis, BH4^• +^ recaptures an electron from this ferrous iron-NO complex, allowing release of gaseous NO [18, 21]. Therefore, BH4 is not consumed during NOS catalysis, but is necessary for both NO synthesis and release. However, BH4 is not required for the initiating step of NO synthesis, which consists of reduction of the heme iron by NADPH-derived electrons so that molecular oxygen may be bound and activated [20]. In the absence of BH4, NADPH consumption and oxygen activation proceed, but are no longer “coupled” to subsequent BH4-dependent L-arginine oxygenation [3–5]. As a result, activated oxygen is released directly from the heme catalytic center as superoxide in a process termed “NOS uncoupling” [3–5]. The addition of increasing concentrations of BH4 effectively reverses NOS uncoupling by proportionally reducing superoxide production and restoring NO synthesis [5]. De novo BH4 synthesis is a central mechanism by which sufficient cellular BH4 concentrations are maintained to support coupled NOS activity.

### BH4 Biosynthesis

BH4 is a heterocyclic nitrogenous molecule derived from guanosine-5′-triphosphate (GTP). The enzymes GTP cyclohydrolase I (GTPCH), 6-pyruvoyl tetrahydrobiopterin synthase (PTPS), and sepiapterin reductase (SPR) catalyze successive steps in BH4 biosynthesis [22]. In most mammalian tissue, GTPCH-regulated conversion of GTP to 7,8-dihydroneopterin triphosphate is the rate-limiting step in this pathway. As a result, BH4 concentrations correlate strongly with GTPCH expression [23]. Transcription-level regulation is primarily responsible for increased BH4 synthesis in endothelial cells [24] and cardiomyocytes [25] in response to proinflammatory cytokine signaling. However, GTPCH also may be regulated post-transcriptionally by protein–protein interaction with GTPCH feedback regulatory protein (GFRP), which mediates feedback inhibition of GTPCH by BH4 [26, 27]. In contrast, disruption of GTPCH-GFRP binding may explain phosphorylation-dependent upregulation of GTPCH activity in response to endothelial shear stress [28, 29]. However, the importance of GFRP regulation may be overshadowed by transcription-level regulation of GTPCH, as evidenced by stable intracellular BH4 concentrations following GFRP overexpression or knockdown in endothelial cells [23].

### BH4: NOS Stoichiometry

By increasing cellular BH4 concentrations, endothelial GTPCH overexpression improves coupled NOS activity in both cells [30] and mouse tissue [10]. Conversely, GTPCH knockdown reduces cellular BH4 concentrations, impairs coupled NOS activity, and increases superoxide production [31]. Furthermore, the ratio of BH4 to eNOS protein correlates strongly with NOS-dependent superoxide production across a range of GTPCH and eNOS expression levels [31]. These findings suggest that absolute cellular BH4 concentrations influence NOS coupling insofar as they reflect the ratio of BH4 to NOS protein. This concept may explain why transgenic overexpression of eNOS (thereby reducing the BH4:eNOS ratio) increases aortic and cardiac superoxide production in mice [32] and accelerates plaque formation in the apolipoprotein E–knockout model of atherosclerosis [33]. Restoration of BH4:eNOS stoichiometry by pharmacological BH4 supplementation [33] or concomitant overexpression of transgenic *GCH1* [32] attenuates superoxide production and disease pathogenesis. The clinical relevance of GTPCH expression is illustrated by the association of a relatively common *GCH1* polymorphism with reduced BH4 production, reduced renal excretion of NO metabolites and evidence of NOS uncoupling in ex vivo vessels [34, 35]. These studies taken together demonstrate that GTPCH expression and activity play an important role in determining cellular BH4, the ratio of BH4 to NOS protein, and NOS uncoupling.

## BH4 Recycling is a Determinant of NOS Uncoupling

### BH4 Oxidation

BH4 may be oxidized to 7,8-dihydrobiopterin (7,8-BH2) or quinoid-dihydrobiopterin (q-BH2) by two distinct mechanisms. BH4 undergoes oxidation to q-BH2 as a result of participating as a cofactor in the enzymatic hydroxylation of phenylalanine, tyrosine, or tryptophan. In the vascular endothelium, BH4 is not consumed by participating in NOS catalysis; instead, the primary mechanism of BH4 consumption is direct oxidation by cellular oxidants. The most abundant product of such oxidation reactions at physiological pH is 7,8-dihydrobiopterin (BH2), which competes with reduced BH4 for binding sites at the NOS oxidase domain [36]. However, because BH2 lacks the ability to supply reductive electrons required for L-arginine oxygenation, the displacement of BH4 by BH2 results in NOS uncoupling [5]. In a physiological setting, high levels of cellular oxidative stress contribute to NOS uncoupling by reducing the abundance of BH4 relative to BH2, thereby decreasing the proportion of NOS protein bound by catalytically active BH4. Thus, NOS uncoupling is influenced by the stoichiometric ratio of BH4 to BH2 as well as the ratio of BH4 to NOS protein [5]. In patients with coronary artery disease, both tissue BH4 concentrations and BH4 to BH2 ratios correlate with superoxide production and acetylcholine-mediated vasodilation [37].

### BH4 Recycling

Dihydrofolate reductase (DHFR) is the enzyme responsible for recycling 7,8-BH2 back to catalytically-active BH4 using reductive electrons from NADPH [38]. Pharmacological DHFR inhibition (methotrexate treatment) or short interfering RNA knockdown of DHFR expression both result in significantly reduced levels of BH4 relative to BH2 [39•, 40]. Although DHFR inhibition does not alter total cellular biopterin concentrations, this reduction in the ratio of BH4 to BH2 is sufficient to increase superoxide production and inhibit L-arginine to L-citrulline conversion, a measure of coupled NOS activity [39•, 40]. Alternately, a similar effect may be achieved by GTPCH knockdown, which decreases cellular concentrations of all biopterins without altering the ratio of BH4 to BH2 [31, 39•, 40]. Concomitant DHFR and GTPCH knockdown increases superoxide production compared to knockdown of either gene alone [39•]. This finding demonstrates that NOS uncoupling is determined by BH4 stoichiometry relative to both BH2 and NOS. The functional interdependence of these ratios may explain why the effects of DHFR inhibition on NOS coupling are significantly stronger in mice with low tissue concentrations of BH4 due to reduced basal GTPCH expression [41]. BH4 recycling may also play a key role in BH4 transport, which is sensitive to DHFR inhibition in vivo [42]. Furthermore, injection of mice with equal parts 6S-BH4 and 6R-BH4 results in tissue accumulation of primarily 6R-BH4 [42]. These findings suggest that the primary mechanism of BH4 transport across the plasma membrane is via oxidation to BH2, an achiral compound, and subsequent reduction to bioactive 6R-BH4 within the cell.

## BH4 Oxidation is a Cause and Consequence of NOS Uncoupling

**Fig. 1:**
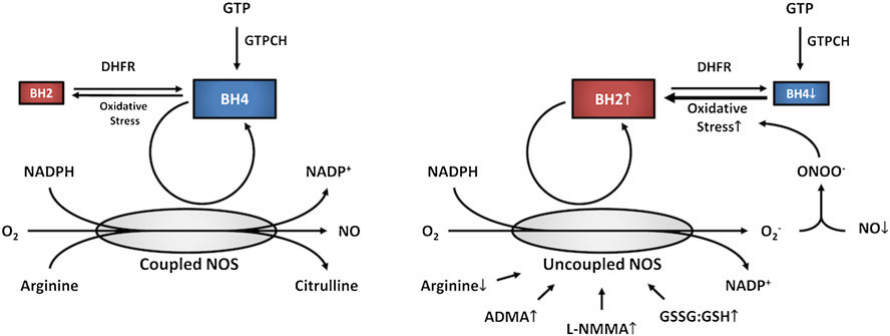
BH4 synthesis, recycling, and oxidation as determinants of NOS uncoupling. *Left* To produce nitric oxide (NO), nitric oxide synthase (NOS) enzymes require the substrates L-arginine and molecular oxygen (O_2_) and the cofactors tetrahydrobiopterin (BH4), reduced nicotinamide adenine diphosphate (NADPH), heme (not pictured), flavin mononucleotide (FMN, not pictured), and flavin adenine dinucleotide (FAD, not pictured). Under normal conditions, BH4 bioavailability is maintained by 1) de novo synthesis from guanosine triphosphate (GTP), in which the rate-limiting step is catalyzed by GTP cyclohydrolase (GTPCH) and 2) dihydrofolate reductase (DHFR)-mediated recycling of 7,8-dihydrobiopterin (BH2), the primary product of nonenzymatic BH4 oxidation. *Right* “Uncoupled” NOS is characterized by production of superoxide (O_2_^−^). NOS uncoupling is promoted by reduced BH4 bioavailability relative to either BH2 or NOS protein. In turn, O_2_^−^ produced by uncoupled NOS reacts with NO, forming peroxynitrite (ONOO^−^), a highly reactive anion that rapidly oxidizes BH4. Therefore, a state of NOS uncoupling is stabilized by self-propagating oxidative stress. In addition to this primary BH4-mediated cycle, additional mechanisms have been shown to promote uncoupling, including reduced arginine bioavailability, high levels of oxidized glutathione (GSSG) relative to reduced glutathione (GSH), or increased concentrations of the endogenous NOS inhibitors L-*N*-monomethylarginine (L-NMMA) and asymmetric dimethylarginine (ADMA)

Superoxide derived from uncoupled NOS or other mechanisms is a critical source of cellular oxidative stress, including BH4 oxidation. Direct BH4 oxidation by superoxide is relatively slow [43], but BH4 is rapidly oxidized to BH2 by peroxynitrite (ONOO^−^) [44, 45], a highly reactive anion produced by the reaction of superoxide with NO [46]. Peroxynitrite also causes significant cellular damage by nitrating amino acid side chain thiol and hydroxyl groups, thereby disrupting enzymatic function. Consequently, the implications of NOS uncoupling include 1) reduced de novo NO production; 2) sequestration of bioactive NO by superoxide anions via peroxynitrite formation; 3) superoxide and peroxynitrite-mediated cellular damage; and 4) peroxynitrite-mediated oxidation of BH4 to BH2, resulting in further propagation of NOS uncoupling. Therefore, NOS uncoupling is a self-reinforcing biochemical state driven by BH4 oxidation (Fig. 1). BH4 oxidation due to uncoupled eNOS-dependent oxidative stress was observed both in vitro [31] and in vivo [32]. In these experiments, uncoupling was induced by increasing eNOS expression under conditions of stable GTPCH expression, effectively reducing the ratio of BH4 to eNOS [31, 32]. With all other variables held constant, the resulting oxidation of BH4 to BH2 can only be attributed to superoxide production by uncoupled NOS.

Cellular superoxide is also produced by a number of other mechanisms, including NADPH oxidase [8, 47, 48•], xanthine oxidase [49], and mitochondrial respiration [50]. During disease pathogenesis, oxidative stress from one or more of these sources could contribute to BH4 oxidation, thereby initiating or maintaining NOS uncoupling [8, 48•]. Additional superoxide resulting from initial BH4 oxidation and NOS uncoupling would promote a cycle of cellular damage, further BH4 oxidation and biochemical stabilization of uncoupled NOS activity (Fig. 1). Interruption of this feed-forward process is a promising strategy for the treatment of a wide range of cardiovascular disease states caused or exacerbated by NOS uncoupling.

## Roles of NOS in Cardiac Physiology and Pathophysiology

### Coronary Blood Flow and Myocardial Function

NO maintains vascular tone by diffusing to the smooth muscle cells in the vascular wall and activating soluble guanylate cyclase to produce cyclic GMP, which in turn modulates calcium influx and smooth muscle contractility. This pathway is the primary mechanism underlying endothelium-dependent vasodilation in response to shear stress or cholinergic stimulation [51]. In the myocardium, NO signaling is therefore an important regulator of both basal blood flow and induced vessel dilation [52]. However, cardiomyocytes also express functional eNOS [53], nNOS [54, 55], and, after cytokine stimulation, iNOS [56]. The major functions of endogenous NOS activity in the myocardium include modulation of cholinergic and β-adrenergic responsiveness [55, 57], stretch-induced Ca^2+^ handling in cardiomyocytes [58], and whole-heart contractility in response to volume loading [59]. BH4 bioavailability plays an important role in supporting myocardial NO production, as evidenced by increased basal heart rate and β-adrenergic responsiveness in mice with reduced GTPCH expression [60]. Further discussion of the complexities of subcellular NOS targeting and physiological roles in the myocardium are available elsewhere [61, 62].

### Overload-induced Heart Failure

One of the first assessments of NOS uncoupling in the context of heart failure was conducted using transaortic constriction in mice as a model of chronic ventricular pressure overload [63]. In the weeks following aortic constriction, untreated wild-type mice develop cardiac hypertrophy, structural remodeling, increased myocardial superoxide production, and, ultimately ventricular dysfunction [63]. Recoupling of NOS via BH4 supplementation reverses these effects and improves cardiac function at 9 weeks of constriction, but similar effects were also achieved by complete knockout of eNOS [63]. These findings suggest that oxidant stress due to eNOS uncoupling is a central mechanism underlying overload-induced heart failure. Follow-up studies using the same model showed that BH4 supplementation can also treat established cardiac dysfunction (4 weeks of transaortic constriction), significantly reversing superoxide production, and improving left ventricular function by 9 weeks after introduction of cardiac overload [64]. Importantly, these studies showed that reversal of cardiac dysfunction cannot be achieved with tetrahydroneopterin or tempol, compounds that exhibit antioxidant properties but are unable to recouple NOS [63, 64].

### Hypertension and Associated Cardiac Dysfunction

The importance of eNOS as a regulator of systemic blood pressure is well-established [65], and the net impact of all three NOS isoforms in maintaining cardiovascular homeostasis was recently assessed in triple knockout mice [66]. Studies in mice with hypertension due to treatment with deoxycorticosterone acetate (DOCA) demonstrated that NOS uncoupling is a critical molecular mechanism involved in the development of hypertension [8, 9]. Aortas harvested from DOCA-treated mice demonstrate evidence of BH4 oxidation, increased superoxide production, and reduced L-arginine to L-citrulline conversion [8]. These effects are eNOS-specific and responsive to BH4 treatment [8] or overexpression of GTPCH [9]. BH4 supplementation also has been shown to recouple NOS and reverse endothelial dysfunction associated with pulmonary hypertension [67] or following infusion with angiotensin II [68] or ACTH [69]. In addition to influencing coronary blood supply, systemic hypertension also determines cardiac loading and represents an important risk factor associated with heart failure. In subsequent in vivo studies, DOCA treatment was shown to result in significant left ventricular diastolic dysfunction associated with BH4 oxidation and NOS uncoupling in the myocardium [70•]. Ventricular diastolic dysfunction can be reversed by treatment with BH4, but not the smooth muscle–targeted vasodilator hydralazine, indicating the therapeutic importance of recoupling NOS as opposed to normalizing blood pressure alone [70•].

### Ischemia/Reperfusion Injury

Cardiac ischemia followed by reperfusion results in significant BH4 oxidation, NOS uncoupling, endothelial dysfunction in coronary arterioles, and impaired coronary blood flow [71, 72]. BH4 supplementation partially inhibits superoxide production and restores NO production in isolated rat hearts [71]. Treatment with BH4 or the BH4 precursor sepiapterin also restores endothelium-dependent vasorelaxation in isolated coronary arterioles and improves ventricular function following ischemia/reperfusion injury [72–74]. However, the ability of BH4 to restore NO production decreases after 30 min and is absent after 90 min of ischemia in isolated rat hearts, suggesting that mechanisms in addition to BH4 depletion also contribute to NOS uncoupling induced by prolonged ischemia [71].

### Atrial Fibrillation

Atrial NO synthesis is reduced in a porcine model of atrial fibrillation [75], and atrial production of superoxide and peroxynitrite is correlated with risk of developing atrial fibrillation in patients undergoing cardiac surgery [76]. In a recent study pairing data from a goat model with data from patients receiving cardiac surgery, NADPH oxidase was found to be an important source of atrial superoxide production in patients with postoperative atrial fibrillation and in goats after 2 weeks of induced fibrillation [48•]. In contrast, BH4 depletion and atrial superoxide production by uncoupled NOS are increased in patients with permanent atrial fibrillation and goats subjected to 6 months of induced fibrillation [48•]. These data suggest that initial oxidative stress from NADPH oxidase may ultimately lead to permanent atrial fibrillation by depleting BH4 and establishing a steady state of NOS uncoupling.

## Additional Mechanisms of NOS Uncoupling

### L-Arginine Bioavailability

The role of L-arginine bioavailability in determining NOS uncoupling was first illustrated by experiments in nNOS-transfected cells that demonstrated increased peroxynitrite-mediated cellular damage under conditions of L-arginine depletion [77]. Subsequent studies in isolated nNOS and eNOS enzymes suggest that the effect of L-arginine depletion depends on NOS isoform, as well as on the presence of BH4. In isolated, BH4-replete nNOS, superoxide production is significantly increased in the absence of L-arginine [78]. This finding suggests that despite the presence of BH4, nNOS ejects activated oxygen as superoxide with greater frequency when L-arginine substrate is not available for further reactions. In the absence of BH4, superoxide production by nNOS is maximal and independent of L-arginine availability [78]. In isolated, BH4-depleted eNOS enzyme, however, superoxide production is in fact stimulated by L-arginine levels [79]. These results suggest that in the absence of BH4, the introduction of L-arginine may induce conformational changes in eNOS that promote electron flow and increase superoxide production if BH4 is not available to couple oxygen activation to L-arginine oxidation. This interpretation is supported by the observation that L-arginine dose-dependently increases NADPH consumption by BH4-depleted eNOS [79].

### Accumulation of Methylarginines

Methylated L-arginine species, including L-*N*-monomethylarginine (L-NMMA) and ω-*N* ^*G*^,*N* ^*G*^-asymmetric dimethylarginine (ADMA), are produced by post-translational methylation of L-arginine residues by protein methyltransferases and liberated by subsequent proteolysis [80]. ADMA and L-NMMA may therefore accumulate due to increased methyltransferase activity [81] and/or inhibition of dimethylarginine dimethylaminohydrolase (DDAH), the enzyme responsible for ADMA catabolism [82]. Both L-NMMA and ADMA inhibit NOS-mediated NO production by competing with L-arginine to bind at the NOS catalytic site [83]. Under conditions of BH4 depletion, however, L-NMMA increases nNOS-mediated superoxide production and both L-NMMA and ADMA increase NADPH consumption and superoxide production by eNOS [78, 79]. In conjunction with BH4 depletion, accumulation of methylarginines may therefore contribute to NOS uncoupling in the context of endothelial or myocardial dysfunction. In support of this hypothesis, a study of patients undergoing coronary artery bypass surgery demonstrated that increased plasma ADMA concentrations correlate with reduced vasodilation and increased superoxide production in ex vivo vessels [84].

### S-Glutathionylation

S-glutathionylation involves the binding of a glutathione tripeptide to the thiol group of an available cysteine residue. In a recent study, S-glutathionylation of two critical eNOS cysteine residues (C689 and C908) significantly inhibited NO production and increased superoxide production [85••]. Furthermore, increased glutathionylation in the aortic rings of oxidant-treated or spontaneously hypertensive rats impaired eNOS-dependent vasorelaxation following acetylcholine stimulation [85••]. The authors of this study speculate that superoxide production following S-glutathionylation occurs at the reductase domain, rather than at the heme catalytic center [85••], although further experimentation is needed to conclusively differentiate these two potential mechanisms of superoxide production.

## Recoupling NOS as a Therapeutic Approach

### L-Arginine Supplementation

L-arginine supplementation has been pursued as a potential approach to restoring NOS-mediated NO production in patients with cardiovascular disease states characterized by endothelial dysfunction. However, completed clinical trials have largely failed to show beneficial effects, and in some cases demonstrated evidence of harm. For example, 3 g of L-arginine per day delivered orally for 6 months resulted in impaired flow-mediated vasodilation, reduced vascular compliance, and low plasma and urinary nitrites and nitrates in a cohort study of patients with peripheral arterial disease [86]. In addition, patients receiving L-arginine also scored lower on treadmill assessments of exercise capacity compared to placebo-treated control patients. Similarly, treatment with 3 g of L-arginine three times daily for 6 months failed to produce a significant effect on ejection fraction or vascular stiffness in a study of patients who suffered myocardial infarction [87]. The group receiving L-arginine treatment did, however, exhibit a higher death rate than the control group leading to closure of enrollment due to safety concerns [87]. Can the general lack of efficacy of L-arginine supplementation be ascribed to the failure to also replete BH4? Based on available biochemical data [78, 79], supplementation of L-arginine under conditions of BH4 depletion is not only insufficient to recouple NOS, but in fact exacerbates NOS uncoupling. Therefore, administration of L-arginine alone would not be expected to improve endothelial function in disease states characterized by BH4 depletion.

### BH4 Supplementation

BH4 supplementation as a therapeutic approach promises the possibility of improving endothelial dysfunction while reducing the risk of hypotension associated with therapies aimed at downstream NO signaling targets. Several physiological experiments have demonstrated improved eNOS-dependent vasodilation following BH4 infusion in individuals with hypertension [88], hypercholesterolemia [89], diabetes [90], or a history of smoking [91]. Early small trials also demonstrated that oral BH4 administration improved forearm blood flow in hypercholestrolemic patients [92] and reduced blood pressure in patients with poorly controlled hypertension [93]. However, these studies were limited in scope, largely due to the instability of BH4 preparations for oral delivery. In 2007, the U.S. Food and Drug Administration approved use of sapropterin, a thermo- and photo-stable, orally available preparation of synthetic 6R-BH4 (trade name: Kuvan [manufactured by Biomarin Pharmaceutical, Inc., Novato, CA]) for the treatment of phenylketonuria (PKU). PKU is a metabolic disorder resulting from a loss-of-function mutation in the BH4-dependent enzyme phenylalanine hydroxylase that is characterized by accumulation of plasma phenylalanine and potentially severe neurological complications. Large phase 3 trials in this patient population demonstrated that sapropterin was well-tolerated and significantly reduced plasma phenylalanine levels by 1 week of therapy [94, 95]. The efficacy and safety profiles demonstrated in these trials resulted in high interest in sapropterin as a potential treatment for cardiovascular disease characterized by endothelial dysfunction. In a study of patients with sickle cell disease, initial results suggested some improvement in reactive forearm vasodilation following sustained treatment with sapropterin. However, trials in patients with systemic or pulmonary hypertension failed to demonstrate significant efficacy, and full data on these and most other registered studies have yet to be published (Table 1).

**Table 1:** Clinical trials of oral sapropterin

Disease state	Intervention	Duration, *wk*	Results	Clinical trial ID
Systemic hypertension	Oral sapropterin, 5 mg/kg, BID	8	No statistically significant effect on SBP vs placebo (http://www.bmrn.com/)	NCT00325962
Pulmonary arterial hypertension	Oral sapropterin, dose escalation every 2 wks from 2.5 to 5 to 10 mg/kg/d + 2 d of 20 mg/kg BID	6	No statistically significant effects vs placebo (http://www.bmrn.com/)	NCT00435331
Sickle cell disease	Oral sapropterin, dose escalation every 4 wks from 2.5 to 5 to 10 to 20 mg/kg/d	16	Improvement of RH-PAT at 8, 12, and 16 wks of treatment. Sapropterin was well tolerated (http://www.bmrn.com/)	NCT00445978
Coronary artery disease	400 mg/d or 700 mg/d	2–6	No statistically significant difference in clinical end points or in dilation or NOS coupling in ex vivo vessels	NCT00423280, see reference [96••]
Peripheral arterial disease	400 mg BID	24	Not reported to date (Start date: Dec 2006; est. completion: Jan 2009)	NCT00403494
Systolic hypertension	Oral sapropterin, 5 mg/kg, BID	8	Not reported (Start date: Dec 2008)	NCT00802893
Chronic kidney disease	Oral sapropterin, 400 mg BID + 400 mg vitamin C BID for the second 6 wks	12	Not reported to date (Start date: May 2008; est. completion: Aug 2009)	NCT00625820
All-cause endothelial dysfunction	Oral sapropterin, 5 mg/kg oral ± 500 mg vitamin C BID	2	Not reported to date (Start date: Sept 2007; est. completion: March 2009)	NCT00532844
Liver cirrhosis, portal hypertension	Oral sapropterin, dose escalation weekly from 5 to 10 mg/kg/d	2	Ongoing (Start date: Oct 2011; est. completion: Jan 2013)	NCT01456286

Synthetic 6R-BH4, Trade name: Kuvan (manufactured by Biomarin Pharmaceutical Inc., Novato, CA)

*BID* twice daily; *SBP* systolic blood pressure; *RH-PAT* reactive hyperemia-peripheral arterial tonometry (a measurement of vasodilation following temporary constriction of the forearm); *NOS* nitric oxide synthase; *est.* estimated.

Despite this general lack of published clinical trial data, a recent report on sapropterin treatment in patients with coronary artery disease provides some mechanistic insight into the limitations of oral BH4 supplementation as a therapeutic approach to treating cardiovascular disease [96••]. In this study, oral sapropterin treatment failed to improve brachial flow-mediated vasodilation, aortic or carotid distensibility, or acetylcholine-induced vasodilation in ex vivo saphenous vein rings [96••]. Plasma and saphenous vein tissue BH4 concentrations were higher in patients receiving sapropterin treatment compared to placebo-treated control patients, but a concomitant increase in BH2 concentrations yielded no significant improvement in the ratio of BH4 to oxidized biopterin species, conversion of L-arginine to L-citrulline, or superoxide production [96••]. Furthermore, exogenous BH4 added to whole blood was rapidly oxidized and incubation of ex vivo saphenous vein rings with exogenous BH4 paradoxically reduced the ratio of BH4 to oxidized biopterins due to accumulation of BH2 [96••]. The ability of BH4 to recouple NOS in patients with cardiovascular disease may therefore be limited by BH4 oxidation, BH2 accumulation, and failure to improve BH4:BH2 ratios. This limitation also has recently been recognized in an animal model of chronic pressure overload-induced heart failure. Following increasing doses of BH4, assessment of myocardial biopterin levels showed that BH2 rose linearly, but BH4 plateaued at higher doses, resulting in BH4:BH2 ratios returning back toward baseline values [97•].

### Indirect Preservation of BH4

Given these limitations of direct BH4 administration, therapeutic interventions aimed at improving or preserving endogenous BH4 bioavailability may represent a viable alternative. For example, the beneficial effects of statins may depend in part on their ability to increase BH4 bioavailability, as has been demonstrated both in vitro [98] and in patients [99]. By improving BH4 recycling and/or reducing oxidant stress, ascorbic acid [44] and 5-methyl-tetrahydrofolate [100] also have been shown to improve BH4 bioavailability and coupled NOS activity.

## Conclusions

Reduced BH4 concentrations relative to cellular BH2 and NOS protein result in the uncoupling of oxygen activation from NO production and promote the generation of superoxide anions instead. Oxidative stress due to uncoupled NOS activity in turn leads to oxidation of BH4 to BH2 and further propagation of NOS uncoupling. NOS uncoupling and BH4 oxidation thus represent a self-perpetuating cycle that ultimately results in impaired NO signaling in addition to cellular damage and inflammation due to increased oxidative stress (Fig. 1). Restoration of BH4 bioavailability therefore represents a promising approach to recoupling NOS for the treatment of cardiovascular disease. However, early clinical trials of oral synthetic BH4 (sapropterin) thus far generally have failed to demonstrate efficacy in the treatment of diseases characterized by endothelial dysfunction (Table 1). These disappointing results may result from failure to improve the ratio of BH4 to BH2 [96••], highlighting the need for further research on cellular and systemic BH4 oxidation, trafficking and recycling. The clinical efficacy of improving BH4 bioavailability also may depend on other biochemical determinants of NOS uncoupling, including cellular concentrations of L-arginine, methylarginines, and oxidized glutathione. Future studies are therefore needed to understand how BH4 bioavailability and these factors interact with one another to determine NOS activity at biochemical, cellular, and systemic levels. Resulting advances could lead to significant improvement in the treatment of the wide range of cardiovascular disease states in which NOS uncoupling is a central pathogenic mechanism.
